# The efficacy of using continuous glucose monitoring as a behaviour change tool in populations with and without diabetes: a systematic review and meta-analysis of randomised controlled trials

**DOI:** 10.1186/s12966-024-01692-6

**Published:** 2024-12-23

**Authors:** Kelli M. Richardson, Michelle R. Jospe, Lauren C. Bohlen, Jacob Crawshaw, Ahlam A. Saleh, Susan M. Schembre

**Affiliations:** 1 School of Nutritional Sciences and Wellness, College of Agriculture, Life and Environmental Sciences, University of Arizona, Tucson, AZ USA; 2 Present Address: Department of Oncology, Lombardi Comprehensive Cancer Center, Georgetown University, 2115 Wisconsin Avenue NW Suite 300, Washington, D.C 20007 USA; 3 Center for Health Promotion and Health Equity, Department of Behavioural and Social Sciences, Brown University School of Public Health, Providence, RI USA; 4 Centre for Implementation Research, Ottawa Hospital Research Institute, Ottawa, ON Canada; 5 Arizona Health Sciences Library, University of Arizona, Tucson, AZ USA

**Keywords:** continuous glucose monitoring, behaviour change, glycaemic control, glycated haemoglobin, precision medicine, precision health, digital health

## Abstract

**Background:**

Continuous glucose monitoring (CGM) holds potential as a precision public health intervention, offering personalised insights into how diet and physical activity affect glucose levels. Nevertheless, the efficacy of using CGM in populations with and without diabetes to support behaviour change and behaviour-driven outcomes remains unclear. This systematic review and meta-analysis examines whether using CGM-based feedback to support behaviour change affects glycaemic, anthropometric, and behavioural outcomes in adults with and without diabetes.

**Methods:**

Ovid MEDLINE, Cochrane Central Register of Controlled Trials, Elsevier Embase, EBSCOhost PsycINFO, and ProQuest Dissertations & Theses Global were searched through January 2024. Eligible studies were randomised controlled trials in adults that implemented CGM-based feedback in at least one study arm compared to a control without CGM feedback. Dual screening, data extraction, and bias assessment were conducted independently. Mean differences in outcomes between intervention and comparison groups were analysed using generic inverse variance models and random effects. Robustness of pooled estimates from random-effects models was considered with sensitivity and subgroup analyses.

**Results:**

Twenty-five clinical trials with 2996 participants were included. Most studies were conducted in adults with type 2 diabetes (*n* = 17/25; 68%), followed by type 1 diabetes (*n* = 3/25, 12%), gestational diabetes (*n* = 3/25, 12%), and obesity (*n* = 3/25, 12%). Eleven (44%) studies reported CGM-affiliated conflicts of interest. Interventions incorporating CGM-based feedback reduced HbA1c by 0.28% (95% CI 0.15, 0.42, *p* < 0.001; *I*^2^ = 88%), and increased time in range by 7.4% (95% CI 2.0, 12.8, *p* < 0.008; *I*^2^ = 80.5%) compared to arms without CGM, with non-significant effects on time above range, BMI, and weight. Sensitivity analyses showed consistent mean differences in HbA1c across different conditions, and differences between subgroups were non-significant. Only 4/25 studies evaluated the effect of CGM on dietary changes; 5/25 evaluated physical activity.

**Conclusions:**

This evidence synthesis found favourable, though modest, effects of CGM-based feedback on glycaemic control in adults with and without diabetes. Further research is needed to establish the behaviours and behavioural mechanisms driving the observed effects across diverse populations.

**Trial registration:**

CRD42024514135.

**Supplementary Information:**

The online version contains supplementary material available at 10.1186/s12966-024-01692-6.

## Introduction

Strategies for disease management and prevention are continuously evolving, with recent efforts shifting from the conventional “one-size-fits-all” model of healthcare towards a more personalised approach. This paradigm shift gained momentum with the launch of the 2015 Precision Medicine Initiative, which focused on tailoring treatment decisions based on an individual’s unique biological, environmental, and lifestyle factors [1]. Since then, the focus has expanded from precision medicine to precision public health, which encompasses personalised approaches to disease prevention and health promotion [2]. One notable application of the precision public health approach is through biological feedback [3]. Biological feedback is a behaviour change technique wherein individuals are provided with their unique biological data to support changes in health behaviours and subsequent health-related outcomes [3, 4]. Its use in disease management and prevention has been rising in popularity since the early 2000s [3], mirroring advancements in wearable biosensing technology [5]. Wearable biosensors offer a promising avenue for delivering personalised biological feedback in real-time, which can empower users to make informed decisions that have a positive impact on their health, particularly when combined with monitoring of related health behaviours. However, little is known about the efficacy of biological feedback as a health intervention tool [3]. 

A prominent example of the implementation of biological feedback as a behaviour change technique is the continuous glucose monitor (CGM) – a small device worn on the abdomen or back of the arm that continuously measures glucose levels. Data from the device is transferred to the user’s mobile device for real-time viewing of current (and retrospective) glucose levels and trends. CGM first became available by prescription in 1999 and was originally intended for people with insulin-dependent diabetes [6]. Over the past two decades, CGM technology and accessibility has improved substantially [6], and there is a growing body of evidence demonstrating the efficacy of CGM for the management of both type 1 (T1DM) and type 2 diabetes (T2DM) [7–10]. In addition to its established use in diabetes management, CGM is increasingly being adopted by individuals without diabetes who are interested in optimising their metabolic health, preventing disease, and improving athletic performance [11, 12]. This growing interest is driven by the availability of consumer-friendly CGM devices and apps that provide real-time glucose monitoring, enabling users to make immediate adjustments to the behaviours associated with their glucose levels, such as their diet and physical activity. The value of the global CGM market is rapidly increasing from approximately USD $5.2 billion in 2021 and is projected to reach USD $16.1 billion by 2030 [13]. This expansion includes increasing interest from health-conscious individuals without diabetes, who can now purchase CGM devices over-the-counter in several countries, including the U.S [14]. 

Despite the growing interest in CGM as a tool to improve or optimise health, little is known about the efficacy of using CGM-based biological feedback to promote health behaviour change. As a first step, we conducted a scoping review of 31 randomised controlled trials (RCTs) to explore the targeted populations, behaviours, outcomes, and protocols of CGM-based biological feedback interventions [15]. Findings from the review revealed that the number of clinical trials implementing CGM-based biological feedback as a means to support behaviour change is rapidly increasing, with the studies being conducted in diverse populations with and without diabetes [15]. Changes in diet and physical activity were commonly targeted behaviours by CGM-based interventions identified in the scoping review, and nearly all studies measured glycated haemoglobin (HbA1c) as an outcome [15]. However, no reviews to date have pooled the effects of these interventions in a meta-analysis. Engler et al. reviewed 13 studies examining CGM as a behaviour change tool, but their search ended in 2019, was limited to T2DM, and they did not pool the results [16]. 

Given that CGMs are now used by individuals with and without diabetes, it is crucial to understand their wide-ranging effects to optimise their use for behaviour change in clinical and public health settings. Therefore, the objective of this systematic review and meta-analysis is to determine whether using CGM-based biological feedback to support health behaviour change, versus comparison groups not using CGM, affects glycaemic, anthropometric, and behavioural outcomes in adults with and without diabetes.

## Methods

This systematic review and meta-analysis followed the Preferred Reporting Items for Systematic Reviews and Meta-Analyses (PRISMA) checklist [17], and was pre-registered in the International Prospective Register of Systematic Reviews (PROSPERO); CRD42024514135 [18]. The Cochrane Handbook for Systematic Reviews of Interventions (version 6.4, 2023) was used to guide this review [19]. 

### Search strategy

In collaboration with a research librarian, a search strategy was devised to capture RCTs that incorporated CGM-based biological feedback to support health behaviour change. In January 2024, the search was conducted within the following electronic databases with no limitation on publication year or language: Ovid MEDLINE, Cochrane Central Register of Controlled Trials, Elsevier Embase, EBSCOhost PsycINFO, and ProQuest Dissertations & Theses Global. The complete search strategy has been published elsewhere [15]. A review of 17 relevant bibliographies was also performed to identify potentially eligible RCTs. EndNote 20 (Clarivate Analytics, Boston, MA) was used to identify and remove duplicate references and retracted articles. The remaining references were imported into DistillerSR^®^ (Evidence Partners; Ottawa, Canada), where they underwent a second round of deduplication to confirm all articles to be screened were unique.

### Study selection

A three-phase screening process was used within DistillerSR^®^ to identify eligible studies. During the first phase of screening, two trained reviewers independently examined the title and abstracts of all studies returned by the search to identify those that were (1) primary analyses of randomised controlled trials, (2) conducted in adults ≥ 18 years, and (3) implemented CGM-based biological feedback in at least one study arm. Those that met these eligibility criteria underwent a second phase of screening, where two trained reviewers retrieved the full-texts of articles and performed double-data extraction to identify studies that implemented CGM-based biological feedback to support health behaviour change. A third phase of screening was employed wherein studies that incorporated CGM-based biological feedback in the comparison arm were additionally excluded.

### Data extraction

A data extraction form was developed within DistillerSR^®^ (Evidence Partners; Ottawa, Canada) and piloted by two trained reviewers prior to use. Extraction items included bibliographic information, participant and intervention characteristics, descriptive statistics (mean, standard deviation) of primary and secondary outcomes for intervention and control groups, and reported conflicts of interest. A complete list of extracted data is presented in Additional file 1. Two independent reviewers performed double-data extraction. Disagreements were discussed between the two reviewers and resolved. If consensus could not be reached, a third trained reviewer made the final determination. If available, previously published study protocols or protocol details from clinical trial registries were reviewed. When necessary, corresponding authors of included studies were contacted to retrieve unreported data.

### Risk of bias assessment

Risk of bias was assessed by two independent reviewers using the Revised Cochrane Risk of Bias Tool for Randomized Controlled Trials (RoB 2) [20]. Each study was judged based on five domains, which were designed to assess risk of bias arising from: (1) the randomisation process, (2) deviations from the intended intervention, (3) missing outcome data, (4) the measurement of the outcome, and (5) the selection of the reported results. Based on the combination of answers from the signalling questions associated with each domain, each study was classified as having a “Low” or “High” risk of bias, or having “Some concerns” for each domain, and overall. Disagreements in classification between the two independent reviewers were discussed and resolved. If the two reviewers could not come to consensus, a third trained reviewer made the final decision.

### Statistical analysis

A meta-analysis was conducted to compare pooled effect sizes between treatment and control subjects for primary and secondary outcomes with suitably comparable intervention and comparison arms. The primary outcome was change in HbA1c. Secondary outcomes included changes in glycaemic variability (time in range (TIR), time above range (TAR)), anthropometry (body weight, body mass index (BMI)), and behavioural outcomes (diet, physical activity). The mean difference between intervention and comparison groups was analysed using generic inverse variance models and random effects. Outcomes reported in different units, such as HbA1c, were converted to the necessary units of analysis using standard formulas to ensure consistency across studies. When extracting data based on between-group differences, correlation coefficients, obtained from publications when reported or taken from previous reviews with a larger pool of trials [21], were used to estimate within-group variability. Additionally, for studies with multiple comparison groups, we combined the results within each study to prevent overrepresentation. HbA1c levels, TIR, and TAR are reported as percentages. The observed reductions represent absolute changes, indicating direct decreases in percentage points, rather than relative changes of the original percentages. For studies with multiple follow-up assessments, the data from the latest available follow-up were included in the meta-analysis. For studies with multiple outcomes (e.g., HbA1c, TIR, TAR, weight, BMI, diet, physical activity), each outcome was analysed in a distinct meta-analysis. This approach minimised the potential of inflation of study weight from multiple outcomes, as each study contributed independently within its respective analysis.

Publication bias was assessed with a funnel plot and Egger’s test [22]. For all analyses, heterogeneity was assessed with the *I*^2^ statistic [23]. For the main outcome of HbA1c, sensitivity analyses were done when an *I*^2^ statistic was more than 50%, which included removing studies with a high risk of bias, influential cases (determined by Baujat plots) [24], and study duration less than 12 weeks. A duration of less than 12 weeks was selected as a threshold since HbA1c reflects average glucose levels over 3 months. The only deviation from the pre-registered protocol was the addition of a sensitivity analysis based on conflict of interest, due to the higher-than-anticipated number of studies with reported conflicts of interest. Subgroup analyses were conducted using pre-specified subgroups, including type of diabetes, severity of diabetes at baseline (HbA1c ≥ 8%, insulin use), duration of the CGM sensor wear, method of CGM feedback, behaviour tracking in the intervention group, whether participants received CGM-based guidance for behaviour change, the timing of CGM-based guidance (i.e., pre- or post-CGM wear), and use of a glucometer in the control group. Glucometer use in the control group was selected as a subgroup because control participants were receiving glucose feedback; however, it was in the form of intermittent finger-pricks, as compared to the intervention group’s CGM which offered continuous glucose feedback. Only those with a sufficient number of studies per subgroup are presented. Analyses were done with R (v4.2.2) using the Meta R package (v7.0.0) [25], Metafor R package (v4.6.0) [26], and Risk-of-Bias VISualization (Robvis) web app [27]. 

## Results

### Search results

5389 records were assessed for eligibility and 5364 were ineligible. Data from 25 RCTs involving a total of 2996 participants were included in the systematic review [28–52]. The PRISMA flow-diagram is presented in Additional file 2.

### Study characteristics

The characteristics of the 25 RCTs included within this review are summarized in Table 1. Studies were conducted across 15 different countries, most commonly the U.S. (*n* = 5/25, 20%), Korea (*n* = 3/25, 12%), and China (*n* = 3/25, 12%). A majority of studies were conducted in individuals with T2DM (*n* = 17/25; 68%) [29–45], followed by T1DM (*n* = 3/25, 12%) [44–46], gestational diabetes (GDM; *n* = 3/25, 12%) [28, 47, 49], and overweight and obesity (*n* = 3/25, 12%) [50–52]. Five (20%) RCTs focused solely on females (4 of which were in pregestational and GDM populations), while the remainder were balanced among male and female participants. Most studies (*n* = 16/25; 64%) had a mean participant age in the mid-to-late 50’s to early 60’s. Studies ranged from 20 to 300 participants (median = 100), with interventions spanning 2–52 weeks (median = 15). All interventions were multi-component, consisting of CGM-based biological feedback in addition to other features such as prospective (*n* = 12/25, 48%) [32–34, 39, 41–43, 47, 49–52] or retrospective (*n* = 15/25, 60%) [28–32, 35, 38–40, 43, 44, 46–49] CGM-based guidance, or tracking of behavioural and/or biological data (*n* = 13/25, 52%) [29, 34, 36–41, 45, 47, 49, 51, 52]. Eleven of the 25 (44%) [28, 30–35, 39, 42, 44, 48] studies reported CGM-affiliated conflicts of interest, primarily with Abbott (*n* = 5/25, 20%) [28, 30, 32, 35, 42], Dexcom (*n* = 5/25, 20%) [28, 30, 32, 34, 39], and Medtronic (*n* = 5/25, 20%) [28, 30, 31, 35, 48]. 

**Table 1:** Characteristics of included randomised controlled trials (*N* = 25)

Bibliographical data (authors, publication year, country)	Participants (*N* randomised; % female; mean age in years ± SD)	Baseline HbA1c (% mean, SD); Insulin use ^b^	Intervention arm	Comparator arm(s)	Outcomes with available data	Measurement timepoint(s) (weeks)	CGM-affiliated conflicts of interest
**Type 2 diabetes (** ***N*** ** = 15)**							
Allen et al., 2008 (USA) [31]	*N* = 52 Female: 52% Age: 57 ± 14	A1c: 8.6 (1.2) Insulin: No	• 3-days of continuous, unblinded CGM • Retrospective CGM-based guidance • Education	• Education	A1c, BMI, PA	8	Minimed Medtronic ^c^
Aronson et al., 2023 (Canada) [32]	*N* = 116 Female: 36% Age: 58 ± 10	A1c: 8.6 (1.1) Insulin: No	• 98-days of continuous, unblinded CGM • Prospective CGM-based guidance • Retrospective CGM-based guidance • Education	• SMBG • Education	A1c, TAR, TIR, weight	16	Abbott ^d, e,f^ Dexcom ^e, f^ Roche ^e, f^
Choe et al., 2022 (Korea) [33]	*N* = 126 Female: 40% Age: 58 ± 12	A1c: 7.9 (0.7) Insulin: Mixed	• 98-days of continuous, unblinded CGM • Prospective CGM-based guidance • CGM training • Education	• SMBG • Tracking: glucose • Education	A1c, weight	12	Daewoong Pharmaceuticals ^c^
Cox et al., 2020 (USA) [34]	*N* = 178 Female: 58% Age: 58 ± 12	A1c: 8.6 (1.6)* Insulin: No	• 35-days of intermittent, unblinded CGM • Prospective CGM-based guidance • Education • Tracking: diet, PA, glucose • CGM alarms • SMBG between CGM wears	Comparison arm 1: • SMBG • SMBG-based guidance • Education • Tracking: diet, PA, glucose Comparison arm 2: • Education (reducing glycaemic excursions) • Tracking: diet, PA, glucose Comparison arm 3: • Education (weight loss) • Tracking: diet, PA, glucose	A1c, BMI, diet, PA	13	Dexcom ^c^
Furler et al., 2020 (Australia) [35]	*N* = 299 Female: 41% Age: 60 ± 10	A1c: 8.9 (1.2) Insulin: Mixed	• 70-days of intermittent, blinded CGM • Retrospective CGM-based guidance • CGM training	• Blinded CGM • Usual care	A1c, TIR	26, 52	Abbott ^c, d,e, f,h^ Bayer ^e, h^ Medtronic ^d, e,f, h^ Roche ^d, e,f, h^
Guo et al., 2023 (China) [36]	*N* = 68 Female: 39% Age:55 ± 14	A1c: 9.0 (2.1)* Insulin: N/R	• 28-days of continuous, unblinded CGM • Real-time CGM-based guidance • CGM training • Education • Tracking: diet, PA, weight • Mobile app	• Education	A1c, BMI	4	N/R
Haak et al., 2017 (France, Germany, UK) [30]	*N* = 224 Female: 33% Age: 59 ± 10	A1c: 8.7 (3.1)* Insulin: Yes	• 182-days of continuous, unblinded CGM • Retrospective CGM-based guidance	• SMBG	A1c, (BMI), TAR, TIR, (weight)	26	Abbott ^c, e,f^ Berlin-Chemie ^e, f^ Dexcom ^e^ Medtronic ^f^ Ypsomed ^e^
Lee et al., 2023 (Korea) [37]	*N* = 294 Female: 34% Age: 56 ± 8	A1c: 7.5 (0.4) Insulin: No	• 28-days of intermittent, unblinded CGM • Real-time CGM-based guidance • Concurrent SMBG • Tracking: diet, PA, weight, blood pressure • Integrated health care platform	Comparison arm 1: • Tracking: diet, PA, weight, blood pressure • Integrated health care platform Comparison arm 2: • Usual care	A1c, weight	12, 24, 36, 48	N/R
Meisenhelder-Smith, 2006 (USA) [38]	*N* = 159 Female: 55% Age: 53 ± 11	A1c: 8.5 (1.3) Insulin: Mixed	• 3-days of continuous, unblinded CGM • Retrospective CGM-based guidance • Tracking: diet, PA, medication, glucose • Education	• Education	A1c	12, 24	N/R
Price et al., 2021 (USA) [39]	*N* = 70 Female: 47% Age: 60 ± 11	A1c: 8.4 (0.7)* Insulin: No	• 30-days of intermittent, unblinded CGM • Prospective CGM-based guidance • Retrospective CGM-based guidance • Tracking: diet, glucose • Education	• SMBG • Education	A1c, TAR, TIR	12	Dexcom ^c, g^
Sato et al., 2016 (Japan) [40]	*N* = 34 Female: 41% Age: 60 ± 9	A1c: 8.2 (1.2)* Insulin: Yes	• 15-days of intermittent, blinded CGM • Retrospective CGM-based guidance • Tracking: diet	• Blinded CGM • SMBG • Tracking: diet	A1c, TAR, TIR	17, 34	N/R
Taylor et al., 2019 (Australia) [41]	*N* = 20 Female: 50% Age: 61 ± 8	A1c: 6.6 (0.9)* Insulin: N/R	• 90-days of continuous, unblinded CGM • Prospective CGM-based guidance • SMBG • CGM training • Tracking: diet, PA, glucose • Education • Diet assignment • PA assignment	• Blinded CGM • SMBG • Tracking: diet, PA, glucose • Education • Diet assignment • PA assignment	A1c, TAR, TIR, weight	12	N/R
Wada et al., 2020 (Japan) [42]	*N* = 100 Female: 31% Age: 58 ± 10	A1c: 7.8 (0.3)* Insulin: No	• 84-days of continuous, unblinded CGM • Prospective CGM-based guidance • CGM training • Education	• Blinded CGM • SMBG • Education	A1c, BMI, TAR, TIR	12, 24	Abbott ^d, e^
Yeoh et al., 2018 (Singapore) [29]	*N* = 30 Female: 57% Age: 63 ± 10	A1c: 9.9 (1.2) Insulin: Mixed	• 14-days of intermittent blinded CGM • Retrospective CGM-based guidance • Tracking: diet, PA	• SMBG • Tracking: glucose	A1c, (TAR), (TIR)	12	N/R
Yoo et al., 2008 (Korea) [43]	*N* = 65 Female: 58% Age: 55 ± 9	A1c: 9.1 (1.0) Insulin: Mixed	• 9-days of intermittent, unblinded CGM • Prospective CGM-based guidance • Retrospective CGM-based guidance • CGM alarms • Education	• SMBG • SMBG-based guidance • Education	A1c, BMI, diet, PA, weight	13	N/R
**Type 1 & 2 diabetes (** ***N*** ** = 2)**							
Cosson et al., 2009 (France) [44]	*N* = 48 Female: 38% Age: 57 ± 5	A1c: 9.17 (0.99)* Insulin: Mixed	• 2-days of continuous, blinded CGM • Retrospective CGM-based guidance	• Blinded CGM • SMBG • SMBG-based guidance	A1c, TAR, TIR	13	A. Menari Diagnostics ^c^
Ruissen et al., 2023 (Netherlands, Spain) [45]	*N* = 226 Female: 36% Age: 51 ± 12	A1c: 7.7 (1.3)* Insulin: Mixed	• 28-days of intermittent, unblinded CGM • Tracking: diet, PA, glucose, medication, mood, weight • Education • App	• Usual care	A1c, BMI, TAR, TIR	37	N/R
**Type 1 diabetes (** ***N*** ** = 1)**							
Zhang et al., 2021 (China) [46]	*N* = 146 Female: 56% Age: 37 ± 20	A1c: 9.1 (1.4)* Insulin: Yes	• 42-days of intermittent, unblinded CGM • Retrospective CGM-based guidance • CGM training • SMBG	• Blinded CGM • SMBG • SMBG-based guidance	A1c, (TIR)	24, 48	N/R
**Pregestational & gestational diabetes (** ***N*** ** = 4)**							
Alfadhli et al., 2016 (Saudi Arabia) [47]	*N* = 130 Female: 100% Age: 33 ± 6	A1c: 5.6 (0.7)* Insulin: Mixed	• 7-days of continuous, unblinded CGM • Prospective CGM-based guidance • Retrospective CGM-based guidance • Tracking: glucose, medication, PA • SMBG	• SMBG	A1c	12	N/R
^a^Murphy et al., 2008 (UK) [48]	*N* = 71 Female: 100% Age: 31 ± 6	A1c: 7.3 (1.2) Insulin: Mixed	• 42-days of intermittent, blinded CGM • Retrospective CGM-based guidance	• Usual care	A1c	34	Medtronic ^c, d^
Voormolen et al., 2018 (The Netherlands) [28]	*N* = 300 Female: 100% Age: 33	A1c: 6.8 (1.0)* Insulin: Yes	• 28-days of intermittent, blinded CGM • Retrospective CGM-based guidance • SMBG	• SMBG	(A1c)	15	Abbott ^e^ Dexcom ^e^ Medtronic ^e^ Roche ^f^ Sensonics ^e, f^
Zhang et al., 2021 (China) [49]	*N* = 110 Female: 100% Age: 32 ± 4	A1c: N/R Insulin: No	• 14-days of continuous, unblinded CGM • Prospective CGM-based guidance • Retrospective CGM-based guidance • Tracking: diet, hypoglycemia, medication, PA	• SMBG • SMBG-based guidance	Diet, PA	2	N/R
**Overweight & obesity (** ***N*** ** = 3)**							
Chekima et al., 2022 (Malaysia) [50]	*N* = 40 Female: 58% Age: 26 ± 5	A1c: 5.2 (0.3)* Insulin: N/A	• 28-days of intermittent, unblinded CGM • Prospective CGM-based guidance • CGM training • Education	• Education	A1c, BMI, Diet, weight	6.4	N/R
Jospe et al., 2020 (New Zealand) [51]	*N* = 40 Female: 55% Age: 42 ± 13	A1c: 6.1 (3.7)* Insulin: N/A	• 28-days of continuous, unblinded CGM • Prospective CGM-based guidance • Tracking: diet	• SMBG • Education • Tracking: diet	A1c, BMI, weight	26	N/R
Schembre et al., 2022 (USA) [52]	*N* = 50 Female: 100% Age: 60 ± 5	A1c: 5.6 (0.4)* Insulin: N/A	• 20-days of continuous, unblinded CGM • Prospective CGM-based guidance • Education • Tracking: weight • Group fitness classes	• Education • Tracking: weight • Group fitness classes	A1c, BMI, weight	16	N/R

*Indicates the baseline HbA1c for the intervention group, in cases when the HbA1c for the complete sample is not available

( ) Outcomes in parentheses indicate that although the outcome was reported in the paper, it was not included in the analysis because it was not in a usable form (e.g., presented graphically or with missing timepoints)

^a^ Indicates pre-gestational diabetes

^b^ “Insulin: No” indicates that no participants used insulin. “Insulin: Yes” indicates that all participants used insulin. “Insulin: Mixed” indicates that some, but not all, participants used insulin

^c^ CGM-affiliated company provided grant funding or devices to the author(s) to support the study

^d^ CGM-affiliated company provided speaker honorarium(s) to author(s)

^e^ CGM-affiliated company provided research support to the author(s) outside the referenced study

^f^ CGM-affiliated company provided personal / consultancy fees to author(s)

^g^ Author(s) are employed at a CGM-affiliated company

^h^ Author(s) serve on an advisory board for a CGM-affiliated company

A1c = glycated haemoglobin; BMI = body mass index; N/A = not applicable; N/R = not reported; PA = physical activity; SMBG = self-monitoring of blood glucose; TAR = time above range; TIR = time in range

Several studies assessed outcomes that could not be included in the analysis because they were not in a usable form (e.g., presented graphically or missing timepoints). Voormolen et al. assessed HbA1c but only reported results graphically [28], Yeoh et al. measured TIR and TAR only in the intervention group [29], and Haak et al. were missing key measures for weight and BMI [30]. 

### Quality of included studies

**Fig. 1:**
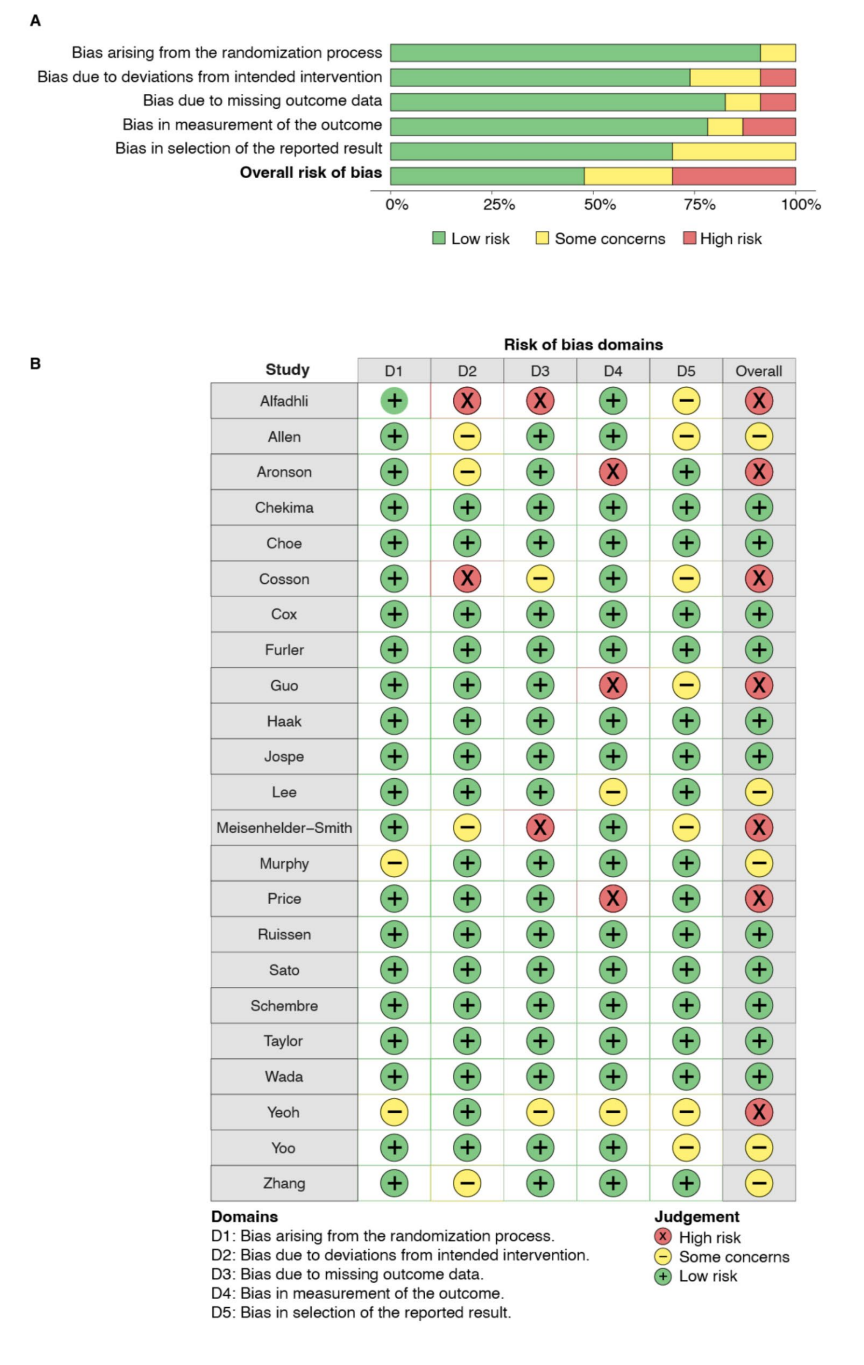
Risk of bias assessment. (**A**) review authors’ judgements about each risk of bias item presented as percentages across all included studies. (**B**) review authors’ judgements about each risk of bias item for each included study

Figure 1 represents the quality of RCTs included for our primary outcome, HbA1c. Of the studies with available HbA1c data, 47.8% (*n* = 11/23) were rated as low risk [30, 33–35, 40–42, 45, 50–52]. Most often, studies were rated as having “some concerns” when bias was present in the selection of the reported result (e.g., no pre-specified plan for analysis). A “high risk” rating was most commonly attributed to studies presenting risk in the measurement of the outcome (e.g., using more than one measurement tool to assess HbA1c among participants).

The funnel plot (Additional file 3) for HbA1c was asymmetrical, indicating possible publication bias or selective reporting within the studies included in this meta-analysis; however, Egger’s test results indicated no significant evidence of small study effects or publication bias (t = -1.284, df = 21, *p* = 0.213). Additionally, the intercept estimate as the standard error approaches zero was -0.075 (95% CI: -0.332 to 0.182), suggesting that any bias due to small study effects is minimal.

### Effectiveness of CGM as a behaviour change tool

### HbA1c

**Fig. 2:**
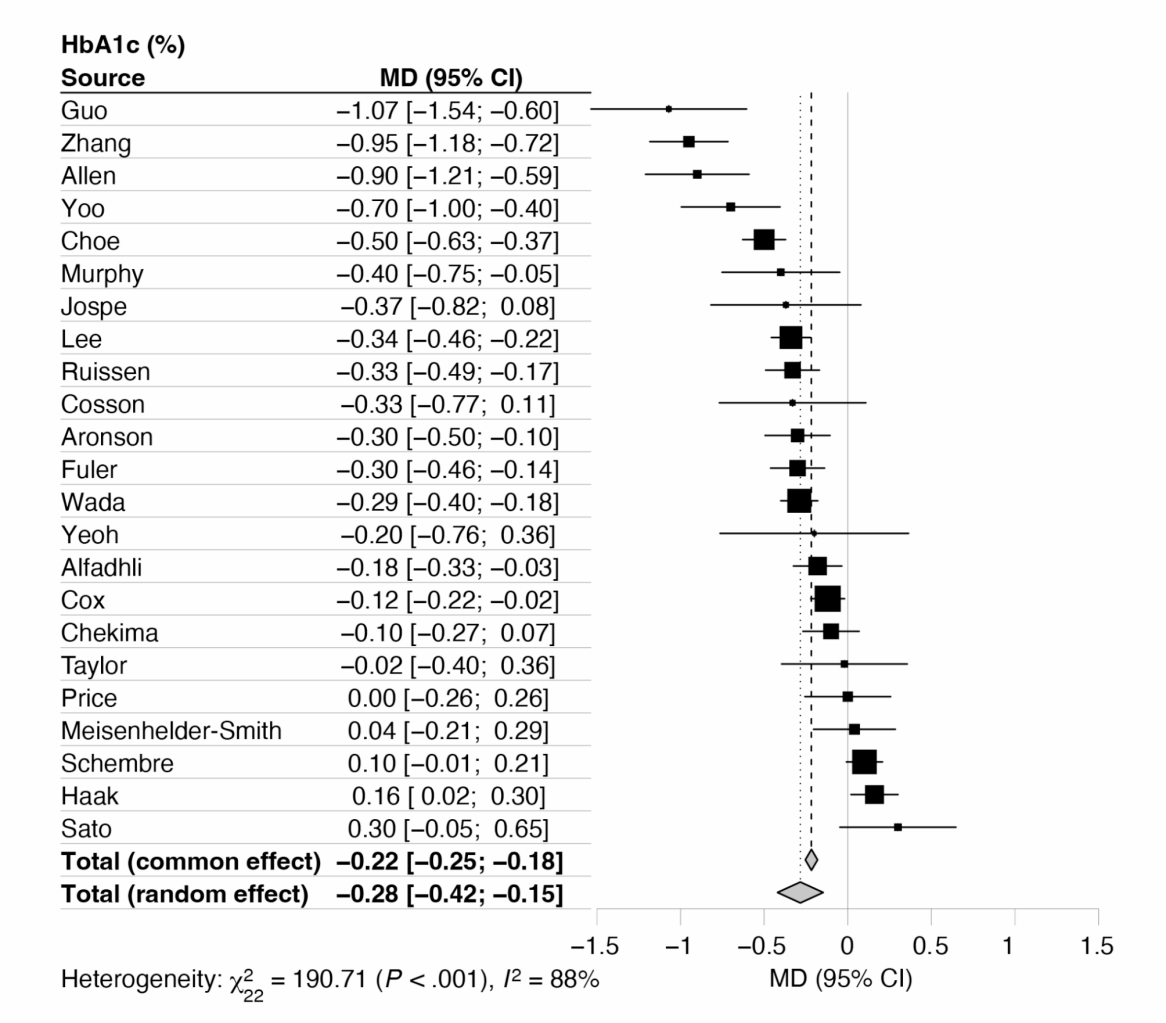
Mean difference in HbA1c (%) between intervention and comparison groups using CGM for behaviour change

**Fig. 3:**
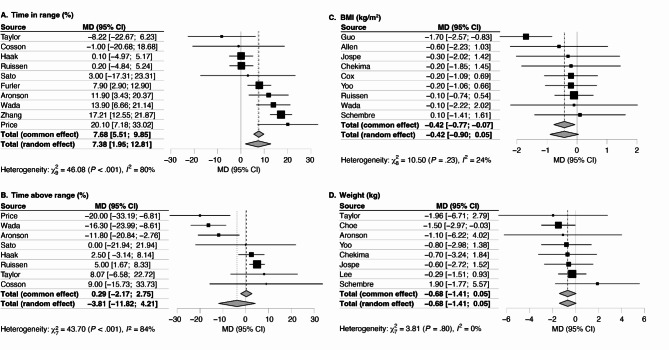
Mean differences between intervention and comparison groups in (**A**) time in range (%), (**B**) time above range (%), (**C**) BMI (kg/m^2^), and (D) weight (kg). BMI: body mass index, MD: mean difference

A summary of key outcome differences between intervention and comparison groups is presented in Table 2. Twenty-three RCTs, including 2355 participants, had available data on the effects of using CGM-based biological feedback on HbA1c (Fig. 3**)** [29–48, 50–52]. HbA1c reduced by 0.28% (95% CI 0.15, 0.42, *p* < 0.001) across intervention arms compared with the comparison arms (Fig. 2). Heterogeneity was high (*I*^2^ = 88%), but sensitivity analyses confirmed the robustness of the main analysis findings, showing consistent mean differences in HbA1c regression across different conditions, though the level of heterogeneity varied (Table 3).

**Table 2:** Summary of key outcome differences between intervention and comparison groups using CGM for behaviour change

Outcome	Studies	Participants (I/C)	I^2^	Mean difference (95% CI)	*p*
HbA1c (%)	23	1135/1220	88.5%	-0.28 (-0.42, -0.15)	< 0.001
TIR (%)	10	608/520	80.5%	7.4 (2.0, 12.8)	0.008
TAR (%)	8	426/326	84.0%	-3.8 (-11.8, 4.2)	0.352
Weight (kg)	8	299/392	0.0%	-0.7 (-1.4, 0.0)	0.066
BMI (kg/m^2^)	9	343/418	18.5%	-0.4 (-0.9, 0.0)	0.080

**Abbreviations**: HbA1c: glycated haemoglobin, BMI: body mass index, TIR: time above range, TAR: time below range, I/C: intervention/comparison

We examined the effect of pre-specified subgroup analyses on HbA1c (Table 4). While there were variations in the mean differences in HbA1c regression across different baseline characteristics and intervention characteristics, none of the differences between subgroups reached statistical significance (*ps* ≥ 0.392). While we had pre-specified that we would perform subgroup analyses for diabetes status and timing of CGM-based guidance, these results were not reported as there was an insufficient number of studies per subgroup to draw conclusions.

**Table 3:** Sensitivity analysis of HbA1c regression

Analysis	Studies	Mean difference			I^2^	
		Mean	95% CI	*p*	Mean	95% CI
Main analysis	23	-0.28	-0.42, -0.15	< 0.001	88.5	84.0, 91.7
Influential cases removed^1^	20	-0.29	-0.42, -0.17	< 0.001	78.0	66.5, 85.6
High risk of bias cases removed^2^	16	-0.29	-0.46, -0.12	< 0.001	91.2	87.3, 93.9
Conflict of interest cases removed^3^	13	-0.32	-0.47, -0.17	< 0.001	85.4	76.6, 90.8
Study duration ≥ 12 weeks^4^	20	-0.24	-0.36, -0.11	< 0.001	87.9	82.7, 91.5

^1^ Three RCTs removed as outliers: Schembre et al., Zhang et al., Haak et al. ^2^ Seven high risk of bias cases: Alfadhli et al., Aronson et al., Cosson et al., Guo et al., Meisenhelder-Smith, Price et al., Yeoh et al. ^3^Eleven RCTs declared a conflict of interest: Allen et al., Aronson et al., Chekima et al., Choe et al., Cosson et al., Furler et al., Haak et al., Jospe et al., Lee et al., Murphy et al., Price et al., Wada et al., Yoo et al. ^4^ Three RCTs were less than 12 weeks in duration: Allen et al., Chekima et al., Guo et al. HbA1c: glycated haemoglobin

**Table 4:** Subgroup analysis of HbA1c regression

Subgroup	Studies	Mean difference	95% CI	*p*	I^2^	Difference between subgroups (*p*)
**HbA1c at baseline**						0.524
< 8%	10	-0.24	-0.36, -0.11	< 0.001	85.8	
≥ 8%	13	-0.33	-0.56, -0.09	0.007	90.6	
**Insulin use at baseline**						0.798
No participants	6	-0.31	-0.53, -0.10	0.004	89.3	
Some / all participants or unspecified	17	-0.27	-0.45, -0.10	0.002	89.9	
**Duration of sensor wear**						0.867
≤ 28 days	13	-0.30	-0.50, -0.09	0.004	87.3	
≥ 29 days	10	-0.27	-0.46, -0.08	0.005	90.4	
**Use of glucometer in control group**						0.622
No	8	-0.34	-0.61, -0.07	0.015	90.0	
Yes	15	-0.26	-0.42, -0.10	0.002	87.8	
**CGM feedback with 2-way communication**						0.891
No	10	-0.30	-0.48, -0.12	0.001	90.8	
Yes	13	-0.28	-0.49, -0.07	0.010	86.3	
**Behaviour tracking in intervention group**						0.392
No	10	-0.35	-0.53, -0.17	< 0.001	88.2	
Yes	13	-0.23	-0.43, -0.03	0.026	88.7	

### Glycaemic variability

TIR was analysed in 10 studies with 1128 participants [30, 32, 35, 39–42, 44–46]. A majority (*n* = 7/10, 70%) reported TIR as 70–180 mg/dL [30, 32, 35, 39, 41, 42, 45]; those that did not reported TIR as 70–150 mg/dL (*n* = 1/10, 10%) [44], 70–140 mg/dL (*n* = 1/10, 10%) [40] or did not specify the lower and upper limits used (*n* = 1/10, 10%) [46]. TIR was increased by 7.4% (95% CI 2.0, 12.8, *p* < 0.008; *I*^2^ = 80.5%) in the intervention group compared to the comparison (Fig. 3A).

TAR was analysed in 8 studies with 752 participants [30, 32, 39–42, 44, 45]. Most (*n* = 5/8, 63%) used 180 mg/dL as the upper limit [30, 32, 39, 41, 42], while one (13%) used 250 mg/dL [45], one (13%) used 150 mg/dL [44], and one (13%) used 140 mg/dL [40]. There was no significant change in TAR between intervention and comparison groups (-3.8% (95% CI -11.8, 4.2, *p* = 0.352); *I*^2^ = 84.0%) (Fig. 3B).

### Anthropometry

Nine studies with 761 participants assessed the effects of CGM-based biological feedback on BMI [31, 34, 42, 43, 45, 50–52], and 8 studies with 691 participants examined the effects on body weight [32, 33, 37, 41, 43, 50–52]. The changes in BMI and weight were not statistically significant (BMI: -0.4 kg/m^2^ (95% CI -0.9, 0.0, *p* = 0.080); weight:  -0.7 kg (95% CI -1.4, 0.0), *p* = 0.066) **(**Fig. 3C and D**)**.

### Behavioural outcomes

Four of the 25 (16%) RCTs assessed diet as an outcome [34, 43, 49, 50], which was measured via food records (*n* = 3/4, 75%) [43, 49, 50] or web-assisted 24-hour dietary recalls (*n* = 1/4, 25%) [34]. Twelve dietary variables were evaluated across the four studies: energy intake (kcals/day), carbohydrate intake (% of total kcals, grams/day, servings/day), fat intake (% of total kcals, grams/day), protein intake (% of total kcals, g/day), cholesterol intake (grams/day), glycaemic index, glycaemic load, and diet control. The most commonly measured aspects of diet were energy intake (kcals/day; *n* = 3/4, 75%) [34, 43, 50], carbohydrate intake (grams/day; *n* = 2/4, 50%) [34, 50], and fat intake (% total kcals; *n* = 2/4, 50%) [43, 50]. Compared to the comparison arms, the intervention arms did not significantly differ in energy intake [43, 50] (*n* = 2; data unavailable for 1 study [34]). However, one study found an increase in fat intake (g/day) and a decrease in carbohydrate intake (g/day) in the intervention arm compared to the control [50]. Given the minimal number of studies that captured each dietary variable, and the variety of measurement tools used, dietary outcomes were not meta-analysed.

Five studies assessed physical activity as an outcome [31, 34, 41, 43, 49], which was measured via self-report (*n* = 2/5, 40%) [43, 49], actigraph (*n* = 2/5, 40%) [31, 41], or Fitbit (*n* = 1/5, 20%) [34]. Nine physical activity variables were measured across the five studies: physical activity time (hours active; minutes active/week), daily step count, sedentary time (% time/day), combined sedentary and light activity (minutes/day), moderate intensity activity (minutes/day), moderate-to-vigorous intensity activity (% time/day), counts/day (defined as the frequency and intensity of movement over a 1-minute interval) and appropriate exercise (undefined). No activity variable was assessed in more than one study; thus, physical activity data could not be pooled. Descriptively, one study assessing minutes of moderate physical activity per day [31], another study assessing minutes of general physical activity per week [43], and a third study assessing appropriate exercise [49], all showed significant increases in the CGM group compared to the control, while one study evaluating combined sedentary and light activity time showed significant decreases [31]. Compared to the control group, one study assessing activity level (counts/day) [31], another study assessing hours active and steps per day [34], and a third study assessing daily percent of time spent sedentary and in moderate-to-vigorous activity [41], did not show significant differences.

## Discussion

Precision public health interventions incorporating CGM-based biological feedback show promise for enhancing health outcomes. While previous research has demonstrated the efficacy of CGM in improving HbA1c levels among adults with T1DM and T2DM [7–10], to our knowledge, this is the first meta-analysis to specifically assess the impact of CGM when used to support behaviour change among adults with and without diabetes. The meta-analysis findings revealed a significant reduction in HbA1c levels by 0.28% and an increase in TIR by 7.4% when using CGM as a behaviour change tool, compared to control conditions without CGM. Although not statistically significant, trends were observed for reductions in weight and BMI with CGM use. There was no significant change in TAR between intervention and comparison groups. Inconsistencies in how dietary and physical activity measures were reported across studies prevented a meta-analysis of these behaviours.

The modest reduction in HbA1c observed in this review is consistent with findings from other meta-analyses [7, 8, 53–57], demonstrating that CGM use, whether implemented to support behaviour change or not (i.e., medication adjustment), effectively lowers HbA1c levels. Reductions of more than 0.3% are considered clinically meaningful by the Food and Drug Administration (FDA) [58] and have been shown to reduce diabetes-related complications [10]. However, clinical impact may vary based on population characteristics and baseline glycaemic control. Our findings were not significantly impacted by participant- nor intervention characteristics, albeit more studies are needed to confirm these findings, particularly in subgroups that have been minimally investigated (e.g., participants without diabetes, inclusion of real-time CGM-based guidance). However, it may be more appropriate to look at other outcome measures in those without diabetes, including glycaemic variability [59, 60] and behaviour changes in response to CGM-based feedback. Notably, 44% of the included studies reported conflicts of interest related to CGM-affiliated companies, which should be considered when interpreting results.

In addition to HbA1c, TIR and TAR outcomes were analysed to assess glycaemic variability. Despite CGM’s ability to measure and quantify these variables, less than half of the included studies reported TIR or TAR. Given that all studies incorporated CGM in the intervention, the exclusion of these data from results represents a missed opportunity to compare changes in glycaemic variability pre- and post-intervention. Based on the available data, this meta-analysis showed that CGM increased TIR by 7.4% compared to the control, which surpasses the threshold of 5% for clinical significance [61] and is consistent with other meta-analyses, which have shown an increase in TIR by 5.6% from baseline following CGM in individuals with T1DM or T2DM [10], and an increase in TIR by 8.6% compared to SMBG in those with T2DM not using insulin [9]. Conversely, no significant change in TAR was observed, which may be due to inconsistent thresholds used to define time above range and the smaller number of studies reporting this outcome. Furthermore, while reductions in BMI and weight were observed, our review showed that changes in these anthropometric variables were not statistically significant following CGM-based biological feedback. This suggests that feedback from CGM may support glycaemic improvements independent of weight change, including among those at lower baseline HbA1c levels and without insulin dependence. These glycaemic benefits may be due to improved dietary intake and increased physical activity. Unfortunately, due to heterogeneity in the reporting of dietary intake and physical activity, we were unable to pool these outcomes, limiting confirmation of specific behavioural mechanisms.

The present review had several strengths. It was the first meta-analysis to include studies focused solely on the use of CGM as a behaviour change tool and was inclusive of adults with and without diabetes. Our results are broadly generalisable given the distribution of men and women, age range (mean age: 26–63 years), and locations (15 countries) observed across the 25 RCTs. Nevertheless, a majority of RCTs (*n* = 15/25; 60%) were conducted in individuals with T2DM, which may limit the generalisability of these results to populations less studied. A serial survey conducted in the US from 2014 to 2020 found that CGM users were more likely to be younger, employed, earning at least $75,000 per year, covered by insurance, and with fewer comorbidities. During the survey period, CGM use increased from 0.4 to 4.1% [62]. More studies are to be expected among populations without diabetes given the increased accessibility and commercialisation of CGM. Additionally, pre-specified sensitivity and subgroup analyses were performed to explore heterogeneity, considering factors such as study duration and risk of bias. However, there were several limitations. High heterogeneity amongst the included studies was a significant concern, potentially affecting the reliability of the findings. Sensitivity analyses were conducted, revealing that changes in HbA1c did not differ significantly across several participant and intervention characteristics, which supports the effects of CGM-based biological feedback on HbA1c. Nonetheless, this approach does not entirely address the issue of heterogeneity, and it remains a limitation that warrants further investigation. Another limitation is that while HbA1c was reported in the majority of studies, other variables were reported in 10 or fewer studies. This indicates a substantial gap in knowledge, suggesting that further research is needed, particularly on behavioural outcomes, to confirm the effect of CGM-based biological feedback on TIR, TAR, weight, BMI, diet, and activity. It also suggests the development of a core outcome set, which would be a minimum set of outcomes to be reported across all CGM-based behavioural intervention studies [63]. Additionally, all interventions were multi-component, differing in the intervention components delivered alongside CGM, which complicates isolating the specific impact of CGM-based feedback. Although subgroup analyses were performed to address the variability in intervention characteristics, such as diet and activity tracking, no significant difference in HbA1c reduction was observed. Variability in comparison groups, which ranged from simple usual care to complex multi-component conditions (e.g., SMBG, education, behavioural tracking), is a recognized challenge in behavioural intervention research and may attenuate CGM effects [64]. Our team is further investigating the impact of these diverse components within intervention and control arms. Understanding comparator group dynamics is essential for accurately assessing CGM-based feedback’s specific effects on behaviour change and health outcomes [65]. 

## Conclusion

In conclusion, this systematic review and meta-analysis suggests that CGM-based biological feedback may support modest improvements in health behaviours that impact glycaemic control in adults with and without diabetes, specifically by reducing HbA1c and increasing TIR. This review highlights several future directions. First, further research on the use of CGM-based biological feedback in populations without diabetes is needed to support the efficacy of this intervention in a variety of populations. Second, the mechanisms by which CGM improves glycaemic measures, such as behaviour change, are poorly understood. Consistency in reporting behavioural measures and the use of high-quality, standardised measurement tools are necessary to compare behavioural outcomes effectively. Lastly, given the multi-component nature of CGM-based biological feedback interventions, research is needed to identify the optimal and most cost-effective combination of intervention components to be delivered alongside CGM. While this review represents a significant step towards understanding the benefits of CGM-based biological feedback on glycaemic, anthropometric, and behavioural outcomes, it also underscores the need for continued investigation to refine and optimise its application across diverse populations.

## Electronic supplementary material

Below is the link to the electronic supplementary material.

Additional file 1

Additional file 2

Additional file 3

## Data Availability

The datasets used and/or analysed during the current study are available from the corresponding author on reasonable request.

## Abbreviations

BCT: Behaviour change technique
BMI: Body mass index
CGM: Continuous glucose monitor
FDA: U.S. Food and Drug Administration
GDM: Gestational diabetes
HbA1c: Glycated haemoglobin
I/C: Intervention/comparison
MD: Mean difference
N/A: Not applicable
N/R: Not reported
PA: Physical activity
PRISMA: Preferred Reporting Items for Systematic Reviews and Meta-Analyses
PROSPERO: International Prospective Register of Systematic Reviews
RCT: Randomised controlled trial
RoB 2: Revised Cochrane Risk of Bias Tool for Randomised Controlled Trials
SMBG: Self-monitoring of blood glucose
T1DM: Type 1 diabetes mellitus
T2DM: Type 2 diabetes mellitus
TAR: Time above range
TBR: Time below range
TIR: Time in range
