# Supplementary material for: The efficacy of using continuous glucose monitoring as a behaviour change tool in populations with and without diabetes: a systematic review and meta-analysis of randomised controlled trials
Source: Int J Behav Nutr Phys Act. 2024 Dec 23;21:145. doi: 10.1186/s12966-024-01692-6 (PMC11668089; doi:10.1186/s12966-024-01692-6)
Supplement: Supplementary file 1 — Additional file 1 [file 12966_2024_1692_MOESM1_ESM.docx]

## **Additional file 1**: Data extracted.

Descriptive data:

1. Bibliographical data (title, authors, year of publication, location, conflicts of interest, funding mechanism)
2. Participant characteristics (population, insulin use, number of participants, % female, mean age, age range, HbA1c eligibility criteria, baseline HbA1c)
3. Primary and secondary outcomes
4. Targeted behaviours
5. Duration of intervention
6. Description of intervention and comparison arms
7. Detailed description of CGM use
   1. Brand and model of CGM
   2. Blinded versus unblinded CGM
   3. Duration of CGM sensor
   4. Number of CGM sensors worn
   5. Duration between CGM wear sessions (if worn more than once)
   6. Communication of CGM results beyond the device (if any)
   7. Who provided CGM feedback (e.g., human, artificial intelligence)
   8. Channel used to provide CGM feedback (e.g., in-person, app, email)
   9. Frequency of CGM feedback
   10. Timing of CGM feedback (e.g., during or after CGM wear)
   11. What (if anything) was personalised based on CGM data (e.g., diet, physical activity)
   12. CGM metrics shared or interpreted (e.g., time in range, mean glucose)

Analysis-specific data:

1. Data extracted for each outcome variable:
   1. Outcome measurement method (eg, laboratory assessment, self-report, not reported)
   2. Quantity of data collection timepoints
   3. Intervention group baseline mean and standard deviation
   4. Intervention group interim mean(s) and standard deviation(s) (if applicable)
   5. Intervention group post-intervention mean and standard deviation
   6. Control group baseline mean and standard deviation
   7. Control group interim mean(s) and standard deviation(s) (if applicable)
   8. Control group post-intervention mean and standard deviation
2. Data extracted specific to Time in Range, Time Above Range, and Time Below Range:
   1. Upper limit
   2. Lower limit
3. Data extracted specific to diet and activity:
   1. Number of days of data (eg, 3, 24-hour diet recalls; 7 days of accelerometery)
   2. Specific aspects of diet and activity being assessed (eg, daily energy intake, minutes of MVPA)
