# Supplementary material for: The efficacy of using continuous glucose monitoring as a behaviour change tool in populations with and without diabetes: a systematic review and meta-analysis of randomised controlled trials
Source: Int J Behav Nutr Phys Act. 2024 Dec 23;21:145. doi: 10.1186/s12966-024-01692-6 (PMC11668089; doi:10.1186/s12966-024-01692-6)

## **Additional file 2**. Preferred Reporting Items for Systematic Reviews and Meta-Analyses (PRISMA) flow diagram.


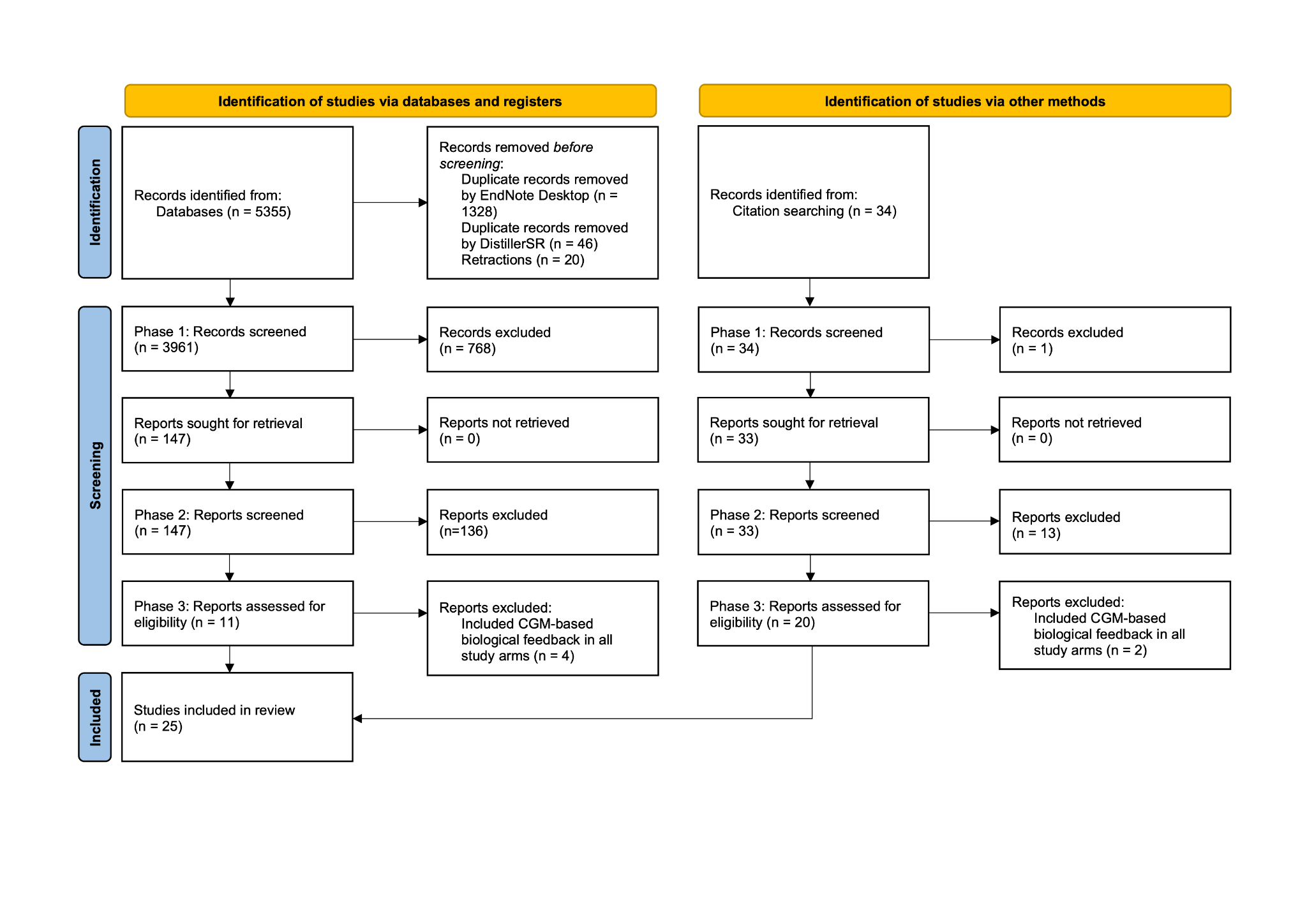

Supplement: Supplementary file 2 — Additional file 2 [file 12966_2024_1692_MOESM2_ESM.docx]
